# An Efficient and Economic Approach for Producing Nanocellulose-Based Aerogel from Kapok Fiber

**DOI:** 10.3390/gels10080490

**Published:** 2024-07-25

**Authors:** Minjie Hou, Qi Wang, Shunyu Wang, Zeze Yang, Xuefeng Deng, Hailong Zhao

**Affiliations:** School of Materials Engineering, Taiyuan Institute of Technology, Taiyuan 030008, China; wangqi20040210@yeah.net (Q.W.); w18526500480@163.com (S.W.); 15296759800@163.com (Z.Y.); niutou0830@163.com (X.D.); 15340657492@163.com (H.Z.)

**Keywords:** kapok, cellulose nanofibers, aerogel, energy consumption, oil absorption

## Abstract

Cellulose nanofibers (NF) were extracted from kapok fibers using TEMPO oxidation, followed by a combination of mechanical grinding and ultrasonic processing. The TEMPO-mediated oxidation significantly impacted the mechanical disintegration behavior of the kapok fibers, resulting in a high NF yield of 98%. This strategy not only improved the fibrillation efficiency but also reduced overall energy consumption during NF preparation. An ultralight and highly porous NF-based aerogel was successfully prepared using a simple ice-templating technique. It had a low density in the range of 3.5–11.2 mg cm^−3^, high compressional strength (160 kPa), and excellent thermal insulation performance (0.024 W m^−1^ K^−1^). After silane modification, the aerogel displayed an ultralow density of 7.9 mg cm^−3^, good hydrophobicity with a water contact angle of 128°, and excellent mechanical compressibility with a high recovery of 92% at 50% strain. Benefiting from the silene support structure, it showed a high oil absorptive capacity (up to 71.4 g/g for vacuum pump oil) and a remarkable oil recovery efficiency of 93% after being reused for 10 cycles. These results demonstrate that our strategy endows nanocellulose-based aerogels with rapid shape recovery and high liquid absorption capabilities.

## 1. Introduction

Aerogels are remarkable materials known for their lightweight nature and high porosity [1,2,3]. They have been developed for diverse applications in thermal insulation [4], sensing [5], catalysis [6], energy harvesting [7] and environmental remediation [8]. The choice of precursors or building blocks for the production of aerogels is pivotal in shaping the microstructure and performance of the final material [9,10]. Aerogels are commonly categorized into three types based on their building blocks: inorganic, organic, and composite aerogels. Among them, inorganic aerogels, derived from silica, are typically brittle, while organic and composite aerogels offer more flexibility [11,12]. However, the synthesis of organic or composite aerogels always involves complex and expensive processes that utilize toxic reagents [13]. Hence, there is an increasing demand for innovative building blocks to facilitate the sustainable and environmentally friendly manufacturing of aerogels.

Cellulose is a highly promising building block for building cellulose aerogels owing to its natural abundance, biocompatibility, and numerous active hydroxyl groups [14,15]. Various cellulose aerogels have been developed based on cellulose nanofibrils (NFs), with the flexible NF aerogel serving as an ideal structural framework for producing multifunctional materials. Jiang et al. [16] prepared a lightweight and mechanically robust NF aerogel using a freezing-thawing technique, resulting in a porous honeycomb structure through NF self-assembling. This aerogel exhibited a high water absorption capacity and good wet resilience and shape recovery properties. Qi et al. [17] utilized high aspect ratio cotton NFs as building blocks to construct a flexible aerogel that can be compressed without structural damage. However, the NF aerogel maintained low elasticity due to the aggregation of nanofibrils into irregular sheets during the ice-induced assembly of the NFs, leading to a non-elastic and unstable architecture of the NF aerogel [18]. Several studies have explored the development of elastic NF aerogels. For instance, Qin et al. [19] fabricated a super-elastic NF aerogel though a two-step ice-templating assembly process. It resulted in an aerogel with a sub-micron fibrous structure that demonstrated excellent compression resilience and rapid shape recovery. Nevertheless, this process is energy-intensive and time-consuming.

Recently, NFs with various structures and surface chemistry have been developed, and discussions have arisen regarding the energy consumption involved in NF production. Eriksen et al. [20] discovered that the energy consumption for homogenizing microfibrillated cellulose could escalate to as high as 70 MWh/t. Kargupta et al. [21] employed a sustainable and scalable froth flotation technology to extract nanofibers from bleached pulp, achieving an energy savings of 2 MWh/t with an NF yield of 54%. TEMPO is a well-known reagent that is widely used for pretreatment of cellulose materials in the laboratory to reduce the energy consumption required for mechanical disintegration [22]. Isogai et al. [23] reported that 2,2,6,6-tetramethylpiperidine-1-oxyl radical (TEMPO)-mediated oxidation can reduce the energy consumption value of fibrillating to 7 MJ/kg, indicating that a negatively charged surface can efficiently impede the entanglement of NFs. However, the influence of pretreatment on the energy consumption level during the manufacturing process has seldom been considered.

In this study, a low density, high strength aerogel based on kapok nanofibers (NFs) was prepared using a directional freeze-drying technique. The method for extracting NFs involved a combination of TEMPO oxidation and mechanical shearing treatment. The fibrillation process using oxidized kapok is scalable, energy-efficient, and economically feasible, offering benefits for the ecological environment and low-carbon development. The aerogel exhibited unique anisotropic properties. Additionally, after silane modification, the aerogel demonstrated enhanced compression resilience, shape recovery, and oil absorption properties, showcasing great potential for practical applications in sustainable oil/water separation.

## 2. Results and Discussion

### 2.1. Preparation of Cellulose Nanofibers (NFs)

**Figure 1:**
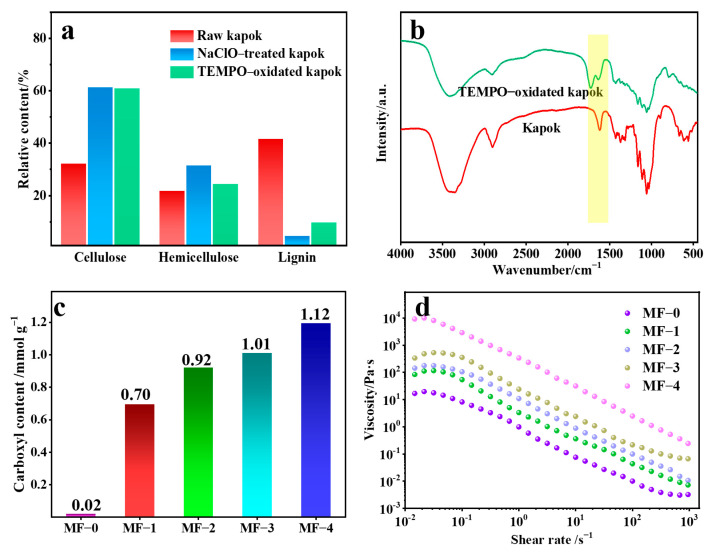
(**a**) The α-cellulose, hemicellulose, and lignin contents of raw kapok, NaClO-treated kapok, and TEMPO-oxidized kapok; (**b**) FTIR spectra of raw kapok and TEMPO-oxidized kapok; (**c**) the carboxyl content of MF samples; (**d**) steady-state viscosity of the MF suspensions.

The choice of cellulose from different sources and scales as building blocks significantly influences the structure and properties of aerogels. Here, natural kapok was chosen as the initial material to produce NFs. The hydrophobic kapok underwent a standard NaClO_2_ treatment to remove its lignin and wax prior to the TEMPO oxidation process [24]. Kapok exhibits a thin cell wall and characteristic hollowness. During the TEMPO oxidation process, carboxyl groups were introduced, making the kapok surface hydrophilic and easy to disintegrate under a grinder. Grinding is a mechanical fibrillation method for NF preparation, achieved by passing the cellulose slurry between static and high-speed rotating grindstones, which applies a shearing stress to the fibers. However, the grinding was inadequate to fibrillate the TEMPO-oxidized kapok fiber, resulting in an interconnected bubble-like structure, as well as inhomogeneous swelling of the kapok [25]. Subsequent sonication treatment disintegrated TEMPO-oxidized microfibers (MFs) into nano-scaled fibrils (NFs). Figure 1a depicts the alterations in cellulose, hemicellulose, and lignin content during the disintegration process. Comparatively, the α-cellulose content of NaClO_2_-treated kapok significantly increased from 32 to 61%, while the lignin content decreased. This demonstrates the effectiveness of NaClO_2_ treatment. Additionally, TEMPO-mediated oxidation converted the C6 hydroxyls of cellulose into C6 carboxyl groups without impacting the α-cellulose content. The chemical structure of kapok before and after TEMPO oxidation was compared by FTIR (Figure 1b). A characteristic band at 1724 cm^−1^ was evident in the spectra of TEMPO-oxidized kapok, indicating the incorporation of a significant number of carboxylate groups [26].

The carboxyl content of the MFs was determined using an alkali-base titration method, and the results are presented in Figure 1c. It was noted that the carboxyl content of the MFs increased from 0.70 to 1.12 mmol g^−1^ as the NaClO additive amount was elevated to 10 mmol g^−1^. Simultaneously, the time required for the complete oxidation of the MFs extended from 0.4 to 3.5 h. This TEMPO oxidation led to MFs with varying charge contents that displayed typical gel-like viscoelastic behavior. As shown in Figure 1d, the steady-state viscosity of the MF suspensions were comprehensively examined. The viscosity reduced gradually with an increase in shear rate from 10^−1^ to 10^3^ s^−1^, showcasing pronounced shear-thinning behavior in all MF suspensions. The networks within the MF suspensions, including inter-fibril physical entanglement, hydrogen bonding, electrostatic repulsion via carboxylate groups, and the interaction between surface charges and water, enabled them to sustain a high viscosity at low shear rates. The viscosity of the MF suspensions consistently rose with a higher NaClO additive amount, with the peak viscosity observed for MF-4, signifying that a greater additive amount of NaClO could introduce more carboxylate groups on the MF surface, enhancing their interaction with water, resulting in more entangled networks and higher viscosity.

### 2.2. Characterizations of NF

**Figure 2:**
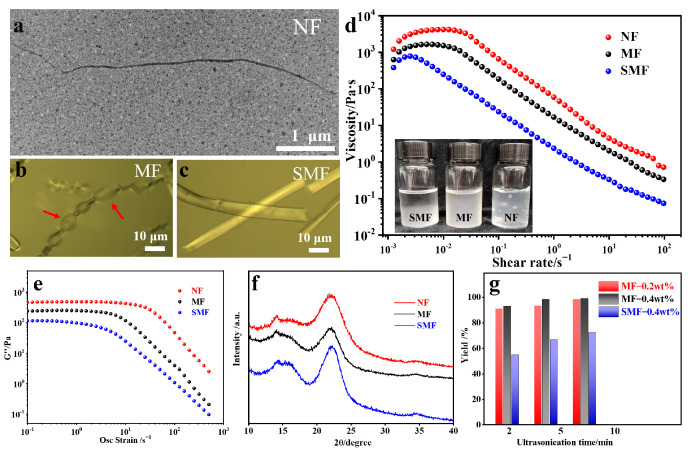
(**a**) TEM image of NF; (**b**,**c**) optical microscopy images of MFs and SMFs; (**d**) steady-state viscosity of the NF, MF, and SMF suspensions at the same concentration of 0.485%; (**e**) loss moduli (G″) versus oscillation strain; (**f**) XRD curves of NFs, MFs, and SMFs; (**g**) Effects of ultrasonication time and solid concentration on NF yields.

After the TEMPO oxidation and defibrillation processes, three types of kapok fibers were prepared and identified as NFs, MFs, and SMFs. NFs exhibited individual fibrils, as depicted in Figure 2a, with a diameter and length of approximately 10.22 nm and 6.86 µm, respectively, and demonstrated a high aspect ratio of around 671 (Figure S1). Microscopic images in Figure 2b,c illustrate the characteristics of MFs and SMFs. It is notable that MFs present a discontinuous bubble shape, distinguishing them from the tubular structure of SMFs, which resembles the heterogeneous swelling behaviors observed during the dissolution of raw cellulose materials [25]. Utilizing the grinding technique, shearing stress was applied to the MF suspension, leading to the partial degradation of the cell wall structure. Among the three samples, the viscosity of NFs at the same shear rate is significantly higher than that of MFs and SMFs (Figure 2d), indicating that numerous nanosized fibrils were stripped from pristine fibers, producing stronger and intertwined networks due to the exposed hydroxyl and carboxyl groups. The surface carboxyl content of the NFs was calculated as 1.20 mmol/g, similar to that of the MFs and SMFs (Figure S2), suggesting that the majority of the surface charge originates from the remaining MFs. Furthermore, their viscoelastic properties were evaluated as a function of oscillation strain (Figure 2e). NFs with a solid content of 0.485% exhibited a higher loss modulus (G″) of 467.4 Pa, which was greater than the values of 240.7 and 115.5 Pa for MFs and SMFs, respectively. These findings were consistent with the rheological results.

The crystallinity was analyzed using X-ray diffraction (XRD) patterns, as illustrated in Figure 2f. All three samples exhibit typical crystalline structure of cellulose I materials, with a calculated degree of crystallinity of 51, 40, and 42% for the NFs, MFs, and SMFs, respectively. The enhanced crystallinity of the NFs can be attributed to the successful removal of hemicellulose and lignin without disrupting its crystalline structure [27]. Interestingly, the grinding process was found to enhance the nano-fibrillation degree of the NFs, thereby minimizing energy usage in the fibrillation processes.

As shown in Figure 2g, the swelled MFs could be exfoliated into NFs with a yield of 9193% within 2 min of sonication (900 W). Extending the sonication time to 10 min resulted in NF yields of 98% and 99% for MF suspensions at concentrations of 0.2 wt% and 0.4 wt%. Compared to previously reported cellulose nanofibers, the NFs were produced at a low cost (see Tables S1–S3). This high production yield is comparable to literature values, and is attributed to two main factors: (1) the surface carboxylic functionalization aided NF exfoliation, and (2) the grinder processing assisted in swelling the thin fiber walls of kapok. Table S2 details the energy consumption previously reported for exfoliating cellulose nanofibers. It was observed that the energy required for the mechanical treatment of MFs is 30.8–83.5 kJ g^−1^, lower than that of tunicate (690.6 kJ g^−1^), wood (247.6 kJ g^−1^), and pulp (102.2 kJ g^−1^). These above average results indicate that our disintegration process is highly efficient and comparable to other mechanical strategies.

### 2.3. Mechanical Performance of NFA Aerogels

**Figure 3:**
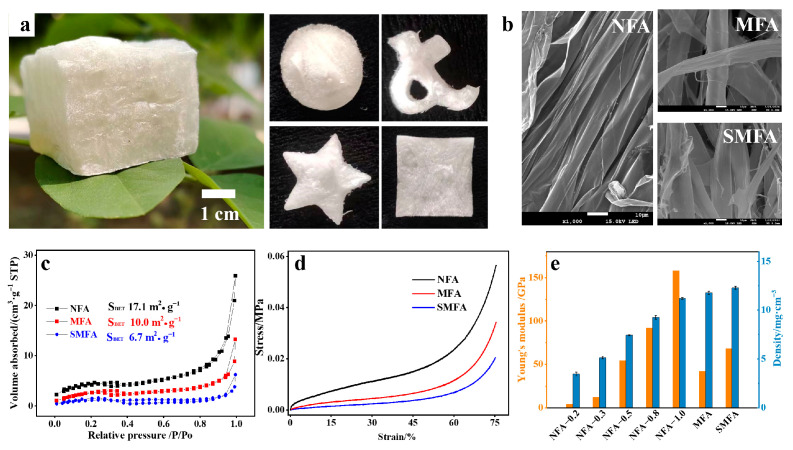
(**a**) Optical image (scale bar, 1 cm) of NFA aerogel, which could be cut into various shapes; (**b**) SEM images (scale bar, 10 µm) of the NFA, MFA, and SMFA aerogels; (**c**) N_2_ adsorption/desorption isotherms of the NFA, MFA, and SMFA aerogels; (**d**) strain and stress curves of the NFA, MFA, and SMFA aerogels; (**e**) the corresponding Young’s moduli and densities.

The NFA aerogel was prepared from an aqueous NF solution using ice templating. NFA aerogels with densities ranging from 3.5 to 11.2 mg cm^−3^ were successfully prepared (refer to Table S4). These aerogels are identified as ultralight and flexible. Additionally, the NFA aerogel can be shaped into various forms depending on the chosen mold (Figure 3a). SEM images shown in Figure 3b display NFA, MFA, and SMFA aerogels at a concentration of 0.2%. Unlike the tubular structure of MFs and SMFs, NFs display an oriented ribbon-like structure, suggesting a more concentrated and extensive assembly caused by ice templating. Figure 3c presents the N_2_ adsorption/desorption isotherms used to investigate the specific surface area of the three aerogels. All three exhibit type II adsorption isotherms without leveling off at a high relative pressure, a typical characteristic of macroporous structures. NFA aerogels have a specific surface area of 17.1 m^2^ g^−1^, higher than that of MFA and SMFA aerogels. This implies that the self-assembly of NFs generates more mesopores, while the morphological structure of MFA and SMFA remains unchanged throughout the ice growth process.

The mechanical properties of the aerogels are presented in Figure 3d. The compressive stress-strain curves of all the aerogels display a typical foam-like property. Three distinct stages were identified based on the curves [28]. First, there is a linear elastic stage at ε < 15%; upon reaching the yield point, it transitioned into a relatively flat section, belonging to the plastic region (15–60%); subsequently, the stress sharply increased during the densification phase at ε > 60%. Due to the alignment of the pore structure parallel to the direction of ice crystal growth, the NFA aerogel exhibited higher stress at the same compression strain of 80%. Moreover, the compressive mechanical performance of the NFA aerogel improved with increasing NF concentrations (Figure S3). The yield stress rose from 0.004 to 0.08 MPa, signifying that the compressive properties resulted from physical interactions and hydrogen bonding between nanofibers. The increased apparent densities could transition NFA aerogels from elastic to rigid characteristic. As illustrated in Figure 3e, the Young’s modulus of NFA-1.0 was obtained as 158 kPa, higher than the 41.8 and 68 kPa of MFA and SMFA aerogels, respectively. Furthermore, in addition to the vertical direction, the mechanical properties of the NFA aerogel in the horizontal direction were also assessed. The ultimate stress of the NFA-0.2 aerogel at 40% strain was determined to be 0.3 kPa, significantly lower than the 2.0 kPa recorded in the vertical direction (Figure S4), implying structural anisotropy.

### 2.4. Thermal Insulation Performance of the NFA Aerogel

**Figure 4:**
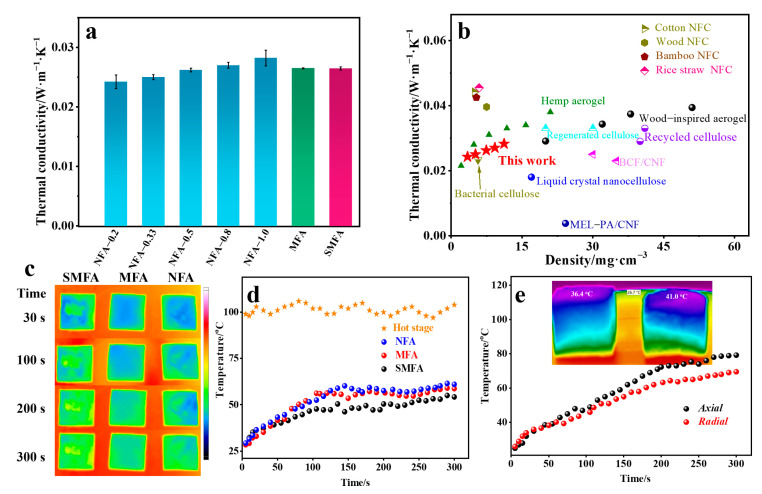
(**a**) Thermal properties of the aerogels; (**b**) comparisons of the thermal conductivity values of cellulose-based aerogels reported in the literature; (**c**) photographs and IR images of aerogels on a 100 °C hot stage; (**d**) the curves of real-time temperature changes of NFA, MFA, and SMFA aerogels (**e**) difference in the heat conduction properties of NFA aerogel in the axial and radial directions.

The thermal insulating performance of the NFA aerogels, which are characterized as a light solid material with a highly porous structure, were evaluated in Figure 4a. The thermal conductivity of all aerogels is below 0.03 W m^−1^ K^−1^, while the thermal conductivity of the NFA aerogel decreases with a reduction in the NF concentration, reaching 0.023 W m^−1^ K^−1^ for the NFA-0.2 aerogel, a value lower than that of air (0.025 W m^−1^ K^−1^) [29]. The low thermal conductivity is comparable to other cellulose-based aerogels, as depicted in Figure 4b. In porous materials, porosity plays a crucial role in determining the thermal conductivity. The higher porosity of the NFA aerogel allows it to be filled with more air, resulting in improved thermal insulating performance. Although the density of the NFA aerogel can reach to 11.2 mg cm^−3^, its thermal conductivity is lower than that of aerogels derived from cotton (0.0442 W m^−1^ K^−1^) [17], wood (0.0396 W m^−1^ K^−1^), and bamboo (0.0425 W m^−1^ K^−1^).

The effectiveness of the NFA aerogel in blocking heat transfer was further demonstrated by placing a piece of aerogel on a hot plate at 100 °C. From the IR images shown in Figure 4c, the hot zone is clearly delineated by the green/red boundary. The color changed from blue to green within the initial 50 s, while the upper surface of the three aerogels maintained a low temperature of approximately 50 °C. Figure 4d illustrates the temperature variations of the three aerogels over a 300-s heating period. The temperature swiftly rose from 25 to 50 °C within 100 s, eventually stabilizing at 56 °C, and close to 54 and 51 °C for MFA and SMFA aerogels, respectively. This suggests that the porous aerogel effectively impedes heat conduction. Moreover, the orientation of the porous structure can influence heat transfer dynamics. The progressive heat transfer through the aerogel in the radial and axial directions are visualized in Figure 4e. The IR thermal gradient is distinctly observable, with the upper surface of the NFA aerogel registering at 36.4 °C in the radial direction and 41.0 °C in the axial direction at the same thickness. The curves suggest that the axial heating rate was greater than that of the radial direction, indicating that the oriented channels within the NFA aerogel contribute to distinct heat conduction properties in the axial and radial directions.

### 2.5. Preparation of NFA-Si Aerogel and the Absorption Performance

**Figure 5:**
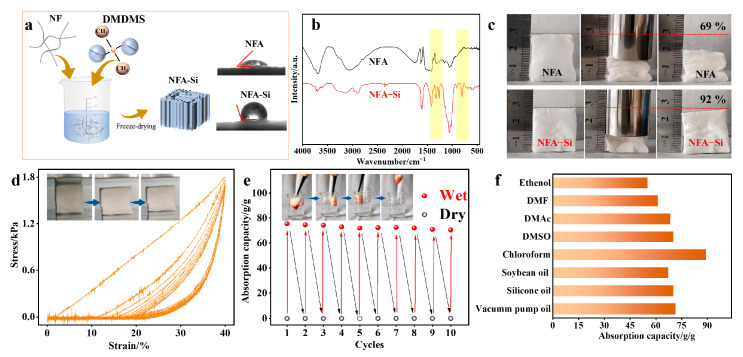
(**a**) The preparation of the NFA-Si aerogel and its water contact angle analysis; (**b**) FTIR spectra of NFA and NFA-Si aerogels; (**c**) the shape recovery performance of NFA and NFA-Si aerogels; (**d**) compression stress-strain curves of NFA-Si aerogel at a strain of 40%; (**e**) cyclic capacity of NFA-Si aerogel to absorb n-hexane; (**f**) the absorption capacities for various oils/organics.

To improve the hydrophobicity and mechanical strength of the NFA aerogel, we achieved a hydrophobic modification through the freezing assembly of NFs and dimethoxydimethylsilane (DMDMS). This process resulted in a uniform hydrophobic fibrous matrix, referred to as NFA-Si (Figure 5a). The resulting NFA-Si aerogel exhibits a low density of 7.9 ± 0.3 mg cm^−3^, with a water contact angle of 128°. In the FTIR spectroscopy results (Figure 5b), characteristic vibrations were observed at 1259, 902, and 804 cm^−1^, corresponding to the structural units of Si-CH_3_, Si-OH, and Si-O-Si, respectively [30]. This confirms the successful polycondensation of hydrolyzed DMDMS sol around the kapok nanofibrous networks, highlighting their robust interactions that strengthen the hybrid aerogel and boost its mechanical performance. As depicted in Figure 5c, the NFA-Si hybrid aerogel showcases remarkable mechanical resilience and flexibility by bouncing back to 92% of its original shape without any significant structural collapse even after enduring a 50% strain. The cyclic compressive stress-stain curves of the NFA-Si aerogel demonstrate excellent elastic behavior over 10 cycles, as shown in Figure 5d. The results indicate that the compressive stress of the NFA-Si aerogel maintains 92% of its initial value after undergoing 10 compressive cycles at a 40% strain.

The excellent reversible compressibility makes the as-prepared aerogels a promising material for oil absorption. The absorption performance of NFA-Si aerogel has been investigated, and the results of cyclical absorption and desorption of n-hexane are summarized in Figure 5e. In each cycle, the NFA-Si aerogel was allowed to absorb a maximum amount of n-hexane, then squeezed by hand to release the absorbed solvent. It can be observed that the absorption capacity shows a slight decrease after 10 cycles, with the adsorption capacity retaining 70.5 g g^−1^, approximately 93% of the initial value. This can be attributed to the structural stability of the NFA-Si aerogel, as well as the combination of a rigid silane network and soft cellulose nanofibers. Additionally, the hydrophobic NFA-Si aerogel demonstrates excellent absorption selectivity in oil/water mixtures. For most organic solvents and oils, the NFA-Si aerogel displays good absorption capacities, as shown in Figure 5f. The absorption capacity of the NFA-Si aerogel towards chloroform reaches 90 g g^−1^, higher than other organic solvents such as DMF (61.2 g g^−1^), DMAc (68.4 g g^−1^), and DMSO (70.2 g g^−1^). In the case of vacuum pump oil, the absorption capacity reaches up to 71.4 g g^−1^, indicating excellent oil adsorption performance. These results demonstrate that this process is an effective and scalable approach to enhance aerogels with increased flexibility and functionalities.

## 3. Conclusions

In summary, the cellulose nanofibers were successfully isolated from biomass kapok though an efficient and cost-effective process that combines TEMPO oxidation and mechanical treatment. The prepared cellulose nanofibers had an average diameter of 8 nm and a crystal structure of nature cellulose I. This strategy resulted in a yield of over 93% with energy consumption below 83.5 kJ g^−1^. The resulting aerogel exhibited anisotropic mechanical and thermal properties, with a high stress of 0.08 MPa along the longitudinal direction, and a low thermal conductivity of 0.024 W m^−1^ K^−1^. After silane modification, the mechanical strength of the NFA-Si aerogel was improved, showing excellent deformation recovery in the radial direction. Furthermore, the NFA-Si aerogel displayed high absorption capacities for a wide range of oil and organic solvents, indicating significant potential for cyclic oil cleanup applications.

## 4. Materials and Methods

### 4.1. Materials

Kapok fibers were from Jiangsu, China, and the crushed kapok fiber was obtained using a fiber cutting machine. 2,2,6,6-tetramethylpiperidine (TEMPO, 98%), sodium bromide (NaBr, 99%) and dimethoxydimethylsilane (DMDMS, 97%) was purchased from Shanghai Aladdin Chemical Regent Co., Ltd. (Shanghai, China). Hydrochloric acid (HCl, 36–38%), sodium hypochlorite (NaClO, 10% available chlorine), acetic acid (99.5%), and other reagents were of laboratory grade and were used without further purification. All water used was purified using a Milli-Q century system (Merck KGaA, Darmstadt, Germany).

### 4.2. Preparation of Micro Kapok Fibers (MFs) and Cellulose Nanofibers (NFs)

**Scheme 1:**
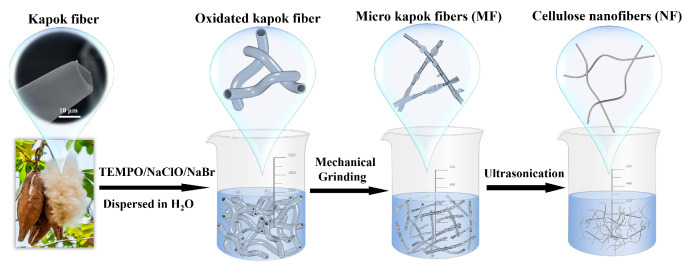
Schematic diagram of the preparation of MFs and NFs.

Micro kapok fibers (MFs): 10 g of the dried kapok fiber was added to a mixed solution (pH = 4) of sodium chlorite (5 g) and acetic acid (1 g) at 80 °C for 4 h. The resulting white fiber was filtered and washed three times with deionized water to eliminate residual chemicals. The TEMPO treatment was performed according to a previously reported method [31]. In accordance with Scheme 1, 1 g of kapok fiber was dispersed in a solution containing TEMPO (0.02 g) and NaBr (0.2 g), with varying amounts of NaClO added for a reaction at pH 10–11. Upon no further NaOH consumption, the TEMPO-oxidized microfibers were obtained and washed. Subsequently, a fiber fibrillation process was conducted, whereby a solution of oxidized kapok fibers (0.5 wt%) was processed using a commercial grinder. The grinding involved five passes, and the resulting MF suspension was collected.

Shattered micro kapok fibers (SMFs): The raw kapok fibers were mechanically crushed before TEMPO oxidation. Then, 1 g of shattered kapok fiber was applied under the same conditions, and was labeled as SMF.

Cellulose nanofibers (NFs): The MF suspension with a concentration of 0.2 or 0.4 wt% were ultrasonicated at 450 W for different durations (2, 5, and 10 min). Then, the precipitate was centrifuged, and the upper suspension of NFs was collected and stored at 5 °C until use. The corresponding volumes of NaClO added for MFs, SMFs, and NFs are listed in Table 1.

### 4.3. Preparation of MFA and NFA Aerogels

The obtained suspension was poured into a pre-customized mold (3 × 3 × 3 cm) and placed on a cold copper plate immersed in a liquid nitrogen bath for unidirectional freezing. The porous aerogel was obtained after removing the ice by freeze-drying for 48 h, and the densities of the aerogels were controlled by the amount of NF (or MF) suspension. These aerogel samples were labeled as NFA-X (Table 2), where X referred to the concentration of the NF suspension. A control SMFA aerogel sample was prepared following a similar procedure.

### 4.4. Preparation of NFA-Si Aerogel

DMDMS (200 µL) was added to a 0.5 wt% NF suspension, and the pH was adjusted to 4.5 with acetic acid to promote the hydrolysis of DMDMS. After stirring at room temperature for 2 h, the suspension was poured into a mold and placed in a liquid nitrogen bath for unidirectional freezing. The composite aerogel was obtained through freeze-drying. To enhance the crosslinking of silane and NFs, the aerogel was baked at 80 °C for 12 h, and the resulting aerogel was named as NFA-Si.

### 4.5. Liquid Adsorption Experiments

To determine the liquid adsorption capacity of the NFA-Si aerogel, various types of organic solvents and oils, including n-hexane, chloroform, ethanol, dimethylformamide (DMF), dimethylsulfoxide (DMSO), soybean oil, silicone oil, and vacuum pump oil were used. Initially, NFA-Si aerogels were weighed (*W*_0_) and then immersed in n-hexane; after adsorption for 20 min, the saturated NFA-Si aerogels were removed, excess n-hexane was eliminated using a filter paper, and then the aerogels were reweighed (*W*_1_). The corresponding adsorption capacity (g/g) was calculated using the following equation:

(1) $$\mathrm{Adsorption}\;\mathrm{capacity}\;(g/g)=\frac{W_{1}-W_{0}}{W_{0}}$$

### 4.6. Characterization

Fourier transform infrared spectroscopy (FTIR) spectra of the samples were recorded on a Bruker Tensor 27 spectrometer with a wave number range from 600 to 4000 cm^−1^.

The optical microscopic images were obtained through an Olympus BX53M optical microscope. The morphology of the aerogels was observed by a field emission scanning electron microscopy (JEOL JSM-7200F, Akishima, Japan).

The content of cellulose, hemicellulose, and lignin was obtained according to the procedures reported in the literature [32]. At least 3 g of an over-dried sample was required, and it was passed through a 1 mm mesh sieve.

The carboxylate content was determined by a conductivity titration method (DDS-11A) and calculated by the following equation:

(2) $$\mathrm{Carboxylate}\;\mathrm{content}\;(\mathrm{mmol}/g)=\frac{(V_{1}-V_{0})\times C_{NaOH}}{m_{0}}$$

where *C_NaOH_* represents the NaOH solution concentration (0.01 mol/L), *V*_1_ and *V*_0_ are the NaOH volumes during titration, and *m*_0_ represents the dried weight of the cellulose sample.

The yield of the NFs was obtained by gravimetric analysis, which was calculated using the following equation:

(3) $$\mathrm{Yield}\;(\%)=\frac{m_{2}}{m_{1}}\times 100\%\;$$

where *m*_1_ represents the weight of raw kapok fiber, and *m*_2_ represents the weight of the NFs, which was obtained from the collected NF suspension after centrifugation.

The rheological properties of the MF suspensions were measured via an ARES-G2 mold rotary rheometer (TA Instruments, New Castle, DE, USA) with a parallel plate geometry of 25 mm in diameter. The steady-state viscosity was determined as a function of shear rate ranging from 0.01 to 1000 s^−1^.

The crystal structure was measured by an X-ray diffractometer (XRD, Billerica, MA, USA) using Ni-filtered Cu K radiation (1.5406 Å) at 40 kV and 40 mA. Scattered radiation was detected in the range of 2θ = 5–40° at a scan rate of 5°/min.

The density of the aerogels was calculated based on the ratio of its weight to volume. The weight (w) of aerogels was measured by an analytical balance, and its structural parameters, such as height, length, and width, were measured by a Vernier caliper. At least 5 measurements were performed for each parameter. Data were expressed as means ± standard deviations (SDs). All statistical analyses were conducted using Microsoft Excel (Version 2406; Microsoft, Redmond, WA, USA). The porosity (%) of aerogels was calculated based on the bulk density and skeletal density of cellulose aerogels using the following equation:

(4) $$\mathrm{Porosity}\;(\%)=\left(1-\frac{\rho_{b}}{\rho_{c}}\right)\times 100\%$$

where *ρ_b_* represents the bulk density of the aerogel samples, and *ρ_c_* represents the skeletal density of cellulose aerogels (1.528 g cm^−3^).

A compression test was performed on an Instron 5969 electronic universal testing machine (Instron Ltd., Norwood, MA, USA). Aerogels of given dimensions (25 × 25 × 12 mm) were prepared, and the test was carried out at 25 °C with a 100 N load cell at a rate of 2 mm/min.

N_2_ desorption was obtained by an ASAP 2460 (Micromeritics, Norcross, GA, USA). The determination of the specific surface area was calculated by the Brunauer–Emmett–Teller (BET) method.

The thermal conductivity measurements of the aerogels were performed using a TPS 2500 S hot disk Thermal Constants Analyzer. A transient plane source (TPS) method was used, and five specimens were measured for each sample.

## Figures and Tables

**Table 1:** The parameters during the preparation process of MFs, SMFs, and NFs.

Sample	Mechanical Crushing	NaClO Pretreatment	NaClO Added in TEMPO Oxidation (mmol)
MF-0	No	Yes	0
MF-1	No	Yes	1
MF-2	No	Yes	3
MF-3	No	Yes	5
MF-4	No	Yes	7
SMF	Yes	Yes	7
NF *	No	Yes	7

* NFs were prepared from the MF-4 suspension with a concentration of 0.2 or 0.4 wt%.

**Table 2:** The corresponding concentration and densities of NFA and MFA aerogels.

Sample	Solid Concentration in Suspension (wt%)	Density (mg cm^−3^)
NFA-0.2	0.2	3.5 ± 0.2
NFA-0.3	0.3	5.1 ± 0.1
NFA-0.5	0.5	7.4 ± 0.1
NFA-0.8	0.8	9.3 ± 0.2
NFA-1.0	1.0	11.2 ± 0.1
MFA	1.0	11.8 ± 0.2
SMFA	1.0	12.3 ± 0.2

## Data Availability

Data will be made available on request.

## Supplementary Materials

The following supporting information can be downloaded at: https://www.mdpi.com/article/10.3390/gels10080490/s1. Figure S1: Diameter distributions and length distributions of NF; Figure S2: Conductometric titration curves of the SMFs, MFs, and NFs, including their carboxylate contents; Figure S3: Strain and stress curves of NFA aerogels with different densities; Figure S4: Compressive capacity of NFA-0.2 aerogel in the axial and radial directions; Table S1: Calculation of the energy, energy consumption, and yield of the NF preparation; Table S2: Comparison of the energy consumption and yields of the NF preparation under different mechanical treatments; Table S3: Comparison of the energy consumption and yields of the biomaterial (pulp and wood) preparation using different methods.
