# Supplementary material for: An Efficient and Economic Approach for Producing Nanocellulose-Based Aerogel from Kapok Fiber
Source: Gels. 2024 Jul 25;10(8):490. doi: 10.3390/gels10080490 (PMC11353854; doi:10.3390/gels10080490)
Supplement: Supplementary file 1 [file gels-10-00490-s001.zip › gels-3101536-supplementary.pdf]

Supplementary Information

Extraction of cellulose nanofibers from kapok with low-cost and silane modified aerogels with improved mechanical and oil absorption properties

Minjie Hou[1,*], Qi Wang, Shunyu Wang[1], Zeze Yang[1], Xuefeng Deng[1] and Hailong Zhao[1]

[1] *School of Materials Engineering, Taiyuan Institute of Technology, Taiyuan 030008, PR China*

*** Corresponding Author**:

E-mail: houminjie0926@126.com (Minjie Hou)

This file includes Supporting Figures S1–S4; Tables S1–S3.

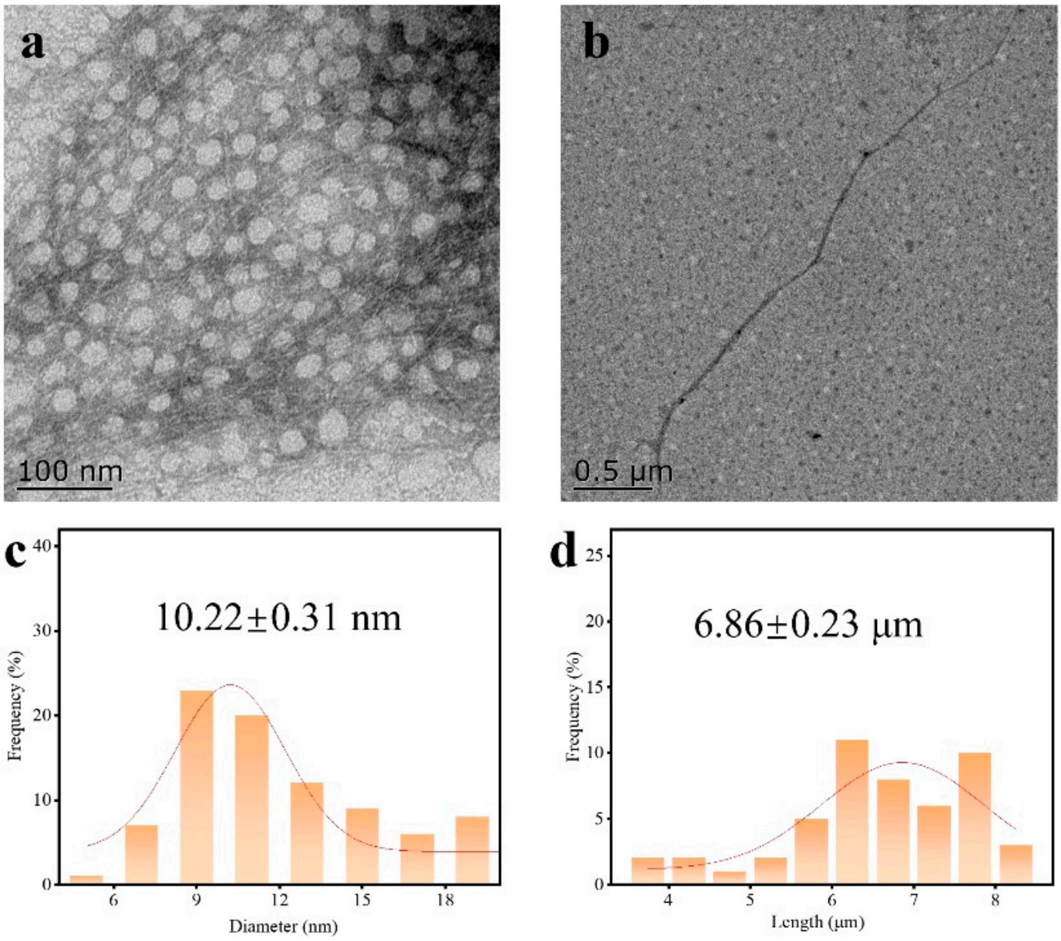

Figure S1. (a, b) TEM image of NF; (c) Diameter distributions of NF, analyzed with “Image J” software; (d) Length distributions of NF, analyzed with “FiberApp” software.

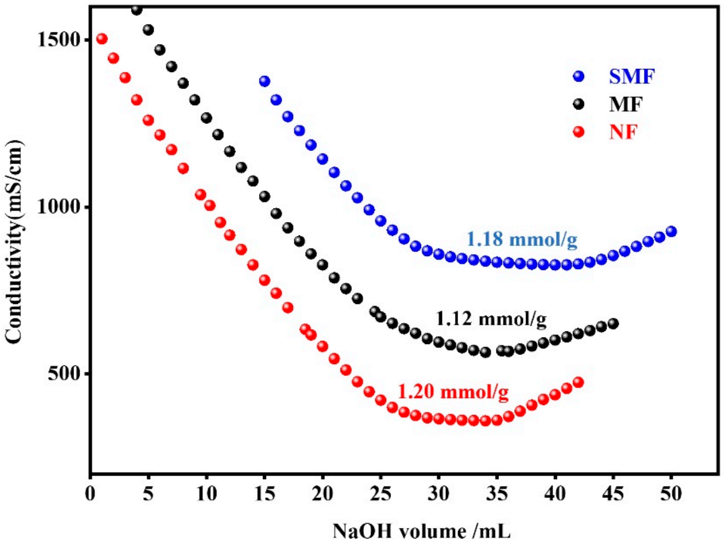

Figure S2. Conductometric titration curves for SMF, MF and NF, including their carboxylate contents.

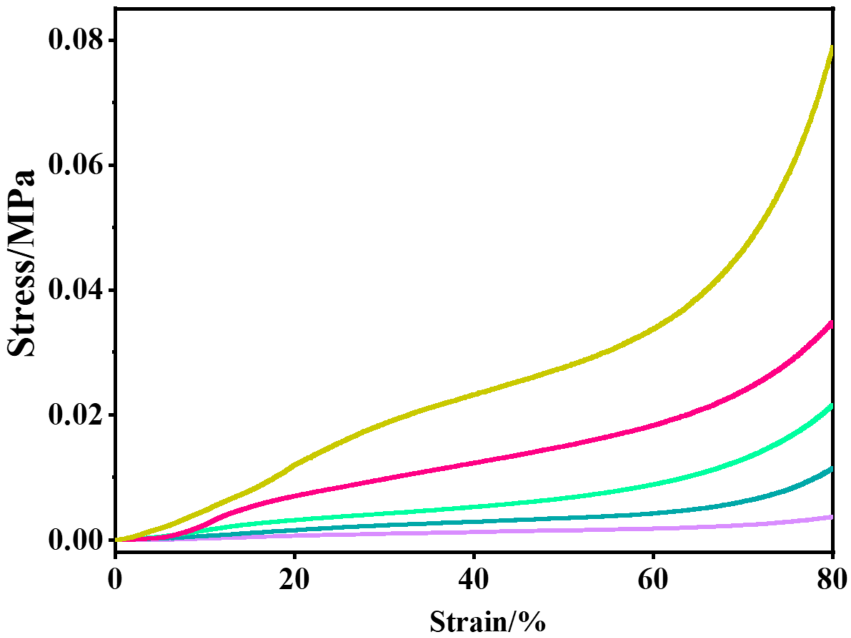

Figure S3. Strain and stress curves for NFA aerogels with different densities.

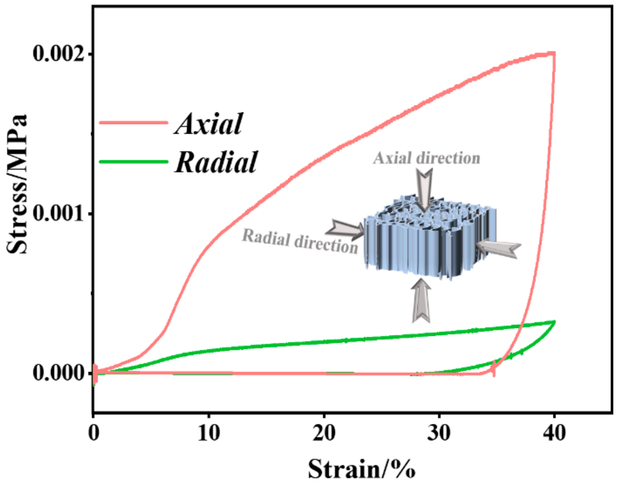

Figure S4. Compressive capacity of NFA-0.2 aerogel in the axial and radial directions.

Table S1. Calculation of the energy, energy consumption and yield of NF preparation.

**Sample**	**Grinding**			**Ultrasonication**			**Energy (kJ)**	**Energy consumption （kJ/g）**	**Yield (%)**
	Power (W)	Time[1] (h)	Energy[2] (kJ)	Power (W)	Time[1] (h)	Energy[2] (kJ)			
1	1100	4.5	17820	900	3.33	10789.2	**28609.2**	**30.8**	**93**
2	1100	4.5	17820	900	7.77	25174.8	**42994.8**	**43.6**	**98.5**
3	1100	4.5	17820	900	20	64800	**82620**	**83.5**	**99**

[1] Time was calculated by the preparation process (treatment of 1 kg raw biomaterial);
[2] Energy consumption = Energy/(1000×Yield), the fibrillation energy was calculated using a power monitor (DL333501C) during the disintegration process.

Table S2. Comparison of the energy consumption and yields of NF preparation under different mechanical treatments.

**Raw material**	**Method**	**Disintegration**	**Energy (kJ)**	**Energy consumption （kJ/g）**	**Yield (%)**	**Ref.**
Tunicate	TEMOP oxidation	Homogenization	490320.0	690.6	71.0	1
Wood	TEMPO oxidation	Homogenization	245160.0	247.6	99	1
Wood	TEMPO&Periodate oxidation	Stirring	900.0	0.978	92	2
Wood	TEMPO oxidation	Homogenization		7	＞90	3
Kapok	TEMPO oxidation	Grinding+Ultrasonic	28609.2	30.8	93	This work
Kapok	TEMPO oxidation	Grinding+Ultrasonic	42994.8	43.6	98.5	This work
Kapok	TEMPO oxidation	Grinding+Ultrasonic	82620	83.5	99	This work
MCC	TEMOP oxidation	Homogenization	11376	11.8	96.1	4
Pulp	TEMPO oxidation	Homogenization	86860.8	102.2	85	5
Ascidiancapsule	TEMPO oxidation	Homogenization	81576	181.3	45	6
Shrimp shell	TEMPO oxidation	Probe sonication	180432	550.1	32.8	6
Squid pen	TEMPO oxidation	Homogenization	120576	172.3	70	6
Bombyx silk	TEMPO oxidation	Homogenization	108576	120.6	90	6

Table S3. Comparison of the energy consumption and yields for the biomaterials (pulp and wood) preparation under different methods.

Raw material	Method	Disintegration		Energy consumption (kJ/g)	Yield (%)	Ref.
		Procedure	Time (h)			
Pulp	TEMPO oxidation	Homogenization	20	102.2	85	5
Pulp		Homogenization	33.4	842.8	21.4	6
Pulp	Carboxymethylation	Homogenization	13.3	95.5	84	6
Pulp	Quaternization	Homogenization	13.3	66.2	86	6
Pulp	Alkali treatment	Homogenization	12	81.8	78.8	6
Pulp	Acid treatment	Ultrasonication	83.3	38.6	88.2	6
Wood	TEMPO oxidation	Homogenization	45.4	248.6	99	1
Wood	Periodate oxidation	Homogenization	18.2	296.4	85	1
Wood	Acid hydrolysis	Ultrasonication	16.7	103.3	60	1

References

1. Zhou M, Chen D, Chen Q, et al. Reversible Surface Engineering of Cellulose Elementary Fibrils: From Ultralong Nanocelluloses to Advanced Cellulosic Materials[J]. Advanced Materials, 2024: 2312220.
2. Gorur Y C, Larsson P A, Wågberg L. Self-fibrillating cellulose fibers: rapid in situ nanofibrillation to prepare strong, transparent, and gas barrier nanopapers[J]. Biomacromolecules, 2020, 21(4): 1480-1488.
3. Isogai A, Saito T, Fukuzumi H. TEMPO-oxidized cellulose nanofibers[J]. nanoscale, 2011, 3(1): 71-85.
4. Salminen R, Reza M, Pääkkönen T, et al. TEMPO-mediated oxidation of microcrystalline cellulose: limiting factors for cellulose nanocrystal yield[J]. Cellulose, 2017, 24: 1657-1667.
5. Isogai A, Hänninen T, Fujisawa S, et al. Catalytic oxidation of cellulose with nitroxyl radicals under aqueous conditions[J]. Progress in Polymer Science, 2018, 86: 122-148.
6. Zhang B, Wang T, Li M, et al. Versatile Deprotonation-Induced Exfoliation and Functionalization of Biological Nanofibrils for Actuation and Fluorescence[J]. ACS Applied Materials & Interfaces, 2024, 16(17): 21665-21671.
